# Evaluation of the Adjuvant Activity of *Herniaria glabra* Saponin-Based Nanoadjuvants

**DOI:** 10.3390/molecules31193523

**Published:** 2026-10-02

**Authors:** Andrey Bogoyavlenskiy, Madina Alexyuk, Pavel Alexyuk, Elmira Omirtayeva, Irina Zaitseva, Yergali Moldakhanov, Elmira Anarkulova, Kuralay Akanova, Timur Kerimov, Aziza Temirbayeva, Vladimir Berezin

**Affiliations:** Research and Production Center for Microbiology and Virology, Almaty 050010, Kazakhstanelya-111@mail.ru (E.A.);

**Keywords:** saponins, immunostimulation, *Herniaria glabra*, nanocomplexes

## Abstract

The increasing use of subunit and recombinant vaccines has created a demand for safe and effective adjuvants capable of enhancing immune responses. Here, we investigated a saponin-rich fraction isolated from *Herniaria glabra* as a source of novel nanoparticulate vaccine adjuvants. The saponins spontaneously self-assembled with lipid components to form stable nanocomplexes with favorable physicochemical characteristics. In a murine immunization model using viral glycoprotein antigens, these nanocomplexes significantly enhanced antigen-specific immune responses, increasing antibody titers by 8- to 16-fold compared with antigen alone. Their adjuvant activity exceeded that of aluminum hydroxide and approached that of *Quillaja saponaria*-derived saponin formulations. The nanocomplexes also induced balanced cellular immune responses comparable to those reported for Quil A-based nanosystems. These findings identify *H. glabra* as a promising alternative source of bioactive saponins and demonstrate the potential of plant-derived nanocomplexes as versatile adjuvant platforms for next-generation vaccines.

## 1. Introduction

Nanotechnology is increasingly transforming modern vaccinology by enabling controlled antigen delivery and modulation of immune responses at the molecular and cellular levels [1,2,3,4]. Nanoparticle-based vaccine platforms can protect antigens from degradation, facilitate their delivery to antigen-presenting cells (APCs), and enhance the magnitude and quality of antigen-specific immune responses. These properties are particularly relevant to subunit and recombinant vaccines, which generally exhibit favorable safety profiles but may have limited intrinsic immunogenicity. Consequently, the development of safe and effective adjuvant systems capable of enhancing and sustaining protective immune responses remains an important priority in vaccine research [2,5]. Among emerging adjuvant strategies, saponin-based nanostructures have attracted considerable attention because of their ability to interact with cholesterol and phospholipids and form highly organized immunostimulatory complexes known as immune-stimulating complexes (ISCOMs) [6,7]. These supramolecular structures can function both as antigen-delivery systems and as immunostimulatory platforms, facilitating antigen uptake and processing while promoting innate and adaptive immune responses. Their particulate organization may also contribute to efficient interaction with immune cells and transport to lymphoid tissues, thereby improving the accessibility of antigens to the immune system. Most studies of ISCOM-based adjuvants have focused on triterpenoid saponins derived from Quillaja saponaria. Quil A, a complex mixture of *Q. saponaria* saponins, can associate with cholesterol and phospholipids to form particulate structures in which saponins contribute both structural and immunostimulatory functions [8,9,10]. Further development of Quillaja-derived adjuvant systems has led to more defined formulations, including Matrix-M™, which have been investigated extensively as vaccine adjuvants and incorporated into licensed vaccine platforms [11,12]. Nevertheless, the relatively limited number of clinically established adjuvant systems highlights the continuing need to investigate alternative immunostimulatory compounds with suitable efficacy, safety, reproducibility, and availability [13,14]. Plant-derived triterpenoid saponins represent an attractive source of potential vaccine adjuvants because of their amphiphilic structures, which facilitate interactions with membrane lipids and can promote the formation of supramolecular assemblies. In addition to their physicochemical properties, many plant saponins possess intrinsic immunomodulatory activity. Although *Q. saponaria* remains an important commercial source of vaccine saponins, the identification of alternative plant-derived saponin mixtures may provide opportunities to develop new adjuvant systems with distinct physicochemical and biological properties [13,14]. Medicinal plants occurring in Kazakhstan represent a potentially valuable source of structurally diverse triterpenoid saponins that may be suitable for the development of supramolecular nanostructures [15,16,17,18]. Despite extensive phytochemical and pharmacological investigations of many medicinal plants, their saponin constituents have been comparatively less explored as components of vaccine adjuvant systems. Investigation of these compounds may therefore expand the range of plant-derived materials available for the development of ISCOM-like nanoparticle formulations. Among medicinal plants of the family *Caryophyllaceae*, *Herniaria glabra L*. is of particular interest because it contains oleanane-type triterpenoid saponins, including bidesmosidic glycosides with pronounced amphiphilic properties [19]. *H. glabra* has traditionally been used for its diuretic, litholytic, anti-inflammatory, other pharmacological properties, and its phytochemical composition and biological activities have been investigated in several studies [20,21,22]. However, despite the reported biological activities of its saponin constituents, their potential to form supramolecular complexes with phospholipids and cholesterol and to function as components of vaccine adjuvant systems has not been sufficiently investigated. The reported anti-inflammatory properties of *H. glabra* do not necessarily preclude the potential use of its saponins as vaccine adjuvant components. The biological effects of saponins are strongly influenced by their molecular structure, concentration, formulation, route of administration, and biological context. Indeed, individual triterpenoid saponins and saponin mixtures may exhibit different, and in some cases apparently contrasting, immunomodulatory effects depending on the experimental conditions. Quillaja-derived saponins, for example, have been extensively investigated for their ability to stimulate immune responses despite the broader range of biological activities associated with these compounds. Therefore, the immunological properties of *H. glabra* saponins need to be evaluated experimentally in the context of an appropriate formulation rather than inferred solely from the reported pharmacological properties of the plant.

In the present study, we investigated the ability of a purified *H. glabra* saponin-rich fraction to form ISCOMMATRIX-like nanocomplexes in combination with phospholipids and cholesterol. The resulting nanocomplexes were characterized with respect to their physicochemical properties, antigen-loading capacity, and cytotoxicity. Their potential adjuvant activity was evaluated using influenza virus glycoproteins as model antigens, with particular emphasis on antigen-specific humoral immune responses in mice. The study was designed to provide initial experimental evidence regarding the suitability of *H. glabra* saponins as components of plant-derived nanoparticle adjuvant systems and to establish a basis for their further investigation as alternative materials for vaccine development.

## 2. Results

### 2.1. Characterization of Nanocomplexes with Herniaria glabra Saponins

Nanocomplexes incorporating *Herniaria glabra* saponins were characterized by dynamic light scattering (DLS) and transmission electron microscopy (TEM). The mean particle size, polydispersity index (PDI), zeta potential, and encapsulation efficiency are summarized in Table 1. Overall, physicochemical parameters of *H. glabra*-based formulations were comparable to those of Quil A-based ISCOMMATRIX, indicating successful formation of structurally similar nanostructures.

TEM analysis confirmed the formation of nanoscale particles with predominantly spherical morphology (Figure 1). The observed particle sizes ranged from approximately 100 to 130 nm, consistent with DLS measurements and indicate good agreement between analytical methods.

The PDI values of approximately 0.35 indicate moderately narrow size distributions, consistent with a relatively uniform nanoparticle population. The negative zeta potential observed for both formulations indicates colloidal stability driven by electrostatic repulsion.

Encapsulation efficiency was high in both systems (>90%), demonstrating efficient incorporation of antigen into the nanostructures.

Importantly, particle sizes in the range of 100–130 nm fall within the optimal window for uptake by antigen-presenting cells, including dendritic cells, which may facilitate efficient lymphatic trafficking and antigen presentation.

Overall, *H. glabra*-derived nanocomplexes exhibited physicochemical characteristics closely comparable to Quil A-based ISCOMMATRIX, supporting their suitability as alternative saponin sources for nanoparticle-based vaccine delivery systems.

The experimental cytotoxicity results are summarized in Table 2.

Under the experimental conditions, the raw *H. glabra* saponin preparation exhibited higher CC_50_ values than Quil A (45.3, 33.41, and 36.4 µg/mL versus 28.13, 15.3, and 18.25 µg/mL, respectively), indicating lower cytotoxicity of the raw *H. glabra* saponins compared with Quil A. We have also clarified that the nanocomplexes exhibit substantially lower cytotoxicity, with CC_50_ values > 100 µg/mL. Encapsulation of saponins within supramolecular nanocomplexes further reduced cytotoxicity across all formulations. Notably, nanocomplexes prepared with *H. glabra* saponins exhibited the highest CC_50_ values among the formulations examined, consistent with enhanced cellular tolerance following nanoencapsulation. Collectively, these findings suggest that *H. glabra* saponins possess a favorable in vitro safety profile and that incorporation into nanostructured formulations may further improve their biocompatibility. Although histopathological evaluation of major organs was not performed, such analyses are generally reserved for comprehensive preclinical toxicity studies rather than initial proof-of-concept investigations focused on adjuvant development.

### 2.2. Induction of Antigen-Specific Humoral Responses by Herniaria glabra-Based Nanocomplexes

The immunogenic potential of the developed nanocomplexes was evaluated using purified influenza virus glycoproteins as model antigens. Animals were immunized with nanocomplexes containing 10 μg of antigen and 2 μg of saponins per dose. Control groups received antigen formulated with aluminum hydroxide, Quil A, or non-complexed saponins.

As shown in Figure 2, influenza virus glycoproteins with *H. glabra*-based nanocomplexes markedly enhanced antigen-specific humoral immune responses, including the production of IgG, IgM, and IgA antibodies. Antibody titers increased approximately 8- to 16-fold compared with antigen alone, demonstrating the pronounced adjuvant activity of the nanocomplex formulation. The magnitude of the response exceeded that induced by aluminum hydroxide and was comparable to that achieved with ISCOMMATRIX-like complexes. multiple IgG subclasses, including IgG1, IgG2a, and IgG2b, whereas IgG3 was not detected (Figure 3). The predominance of IgG2a and IgG2b, together with the simultaneous induction of IgG1, suggests the activation of both Th1- and Th2-associated immune pathways, consistent with the ability of saponin-based adjuvants to promote balanced adaptive immune responses. Because the immunological significance of the absence of detectable IgG3 remains uncertain, additional studies examining cytokine production and cellular immune responses will be required to further characterize the immune profile induced by these nanocomplexes.

Overall, *H. glabra*-based nanocomplexes elicited substantially stronger antibody responses than aluminum hydroxide and achieved immunogenicity comparable to that of established ISCOMMATRIX-like formulations. Together with their favorable physicochemical characteristics and low in vitro cytotoxicity, these findings support the further investigation of *H. glabra*-derived nanocomplexes as promising plant-based adjuvant systems for nanoparticle vaccine formulations.

The results are the average results from six separate mice. Different lowercase letters indicate statistically significant differences among groups according to one-way ANOVA, followed by Tukey’s multiple comparison test (*p* < 0.05). Groups sharing at least one letter are not significantly different.

**Figure 3:**
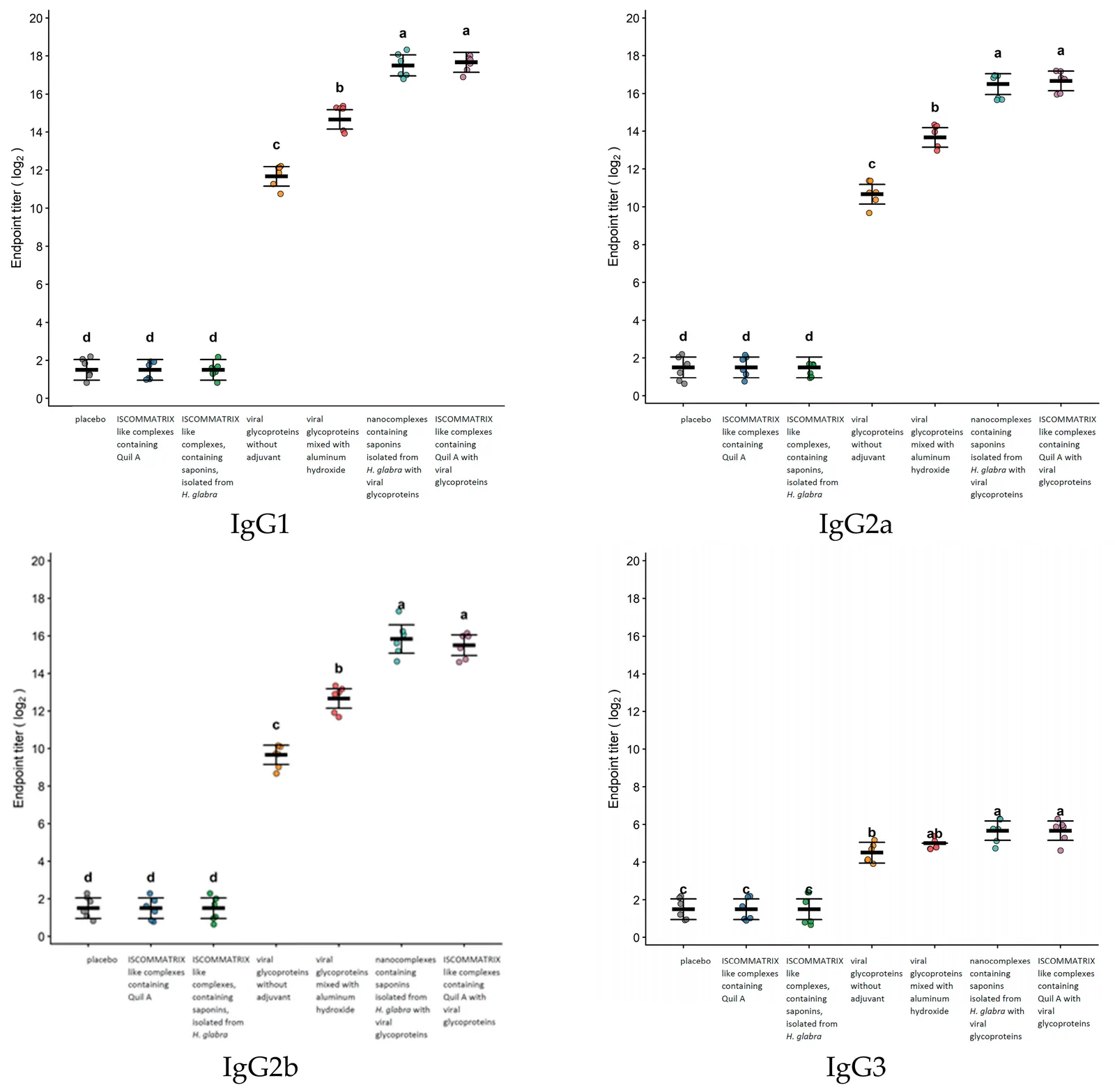
Induction of different IgG classes with the nanocapsulated adjuvant containing saponins isolated from *H. glabra* in a model of human influenza virus glycoproteins, strain A/Almaty/8/98 (H3N2).

The data presented are the average results from six separate mice. Different lowercase letters indicate statistically significant differences among groups according to one-way ANOVA followed by Tukey’s multiple comparison test (*p* < 0.05). Groups sharing at least one letter are not significantly different.

### 2.3. Induction of Virus-Neutralizing Antibodies

The ability of the nanocomplexes to induce functional neutralizing antibodies was evaluated following immunization with influenza A (H3N2) virus glycoproteins. Virus-neutralizing antibodies represent an important correlate of protective immunity because they inhibit viral entry into host cells and prevent infection.

As shown in Figure 4, all tested formulations elicited detectable virus-neutralizing antibody responses. Notably, *H. glabra*-based nanocomplexes induced neutralizing antibody titers comparable to those achieved with ISCOMMATRIX-like formulations and substantially higher than those elicited by aluminum hydroxide or non-complexed saponins.

**Figure 4:**
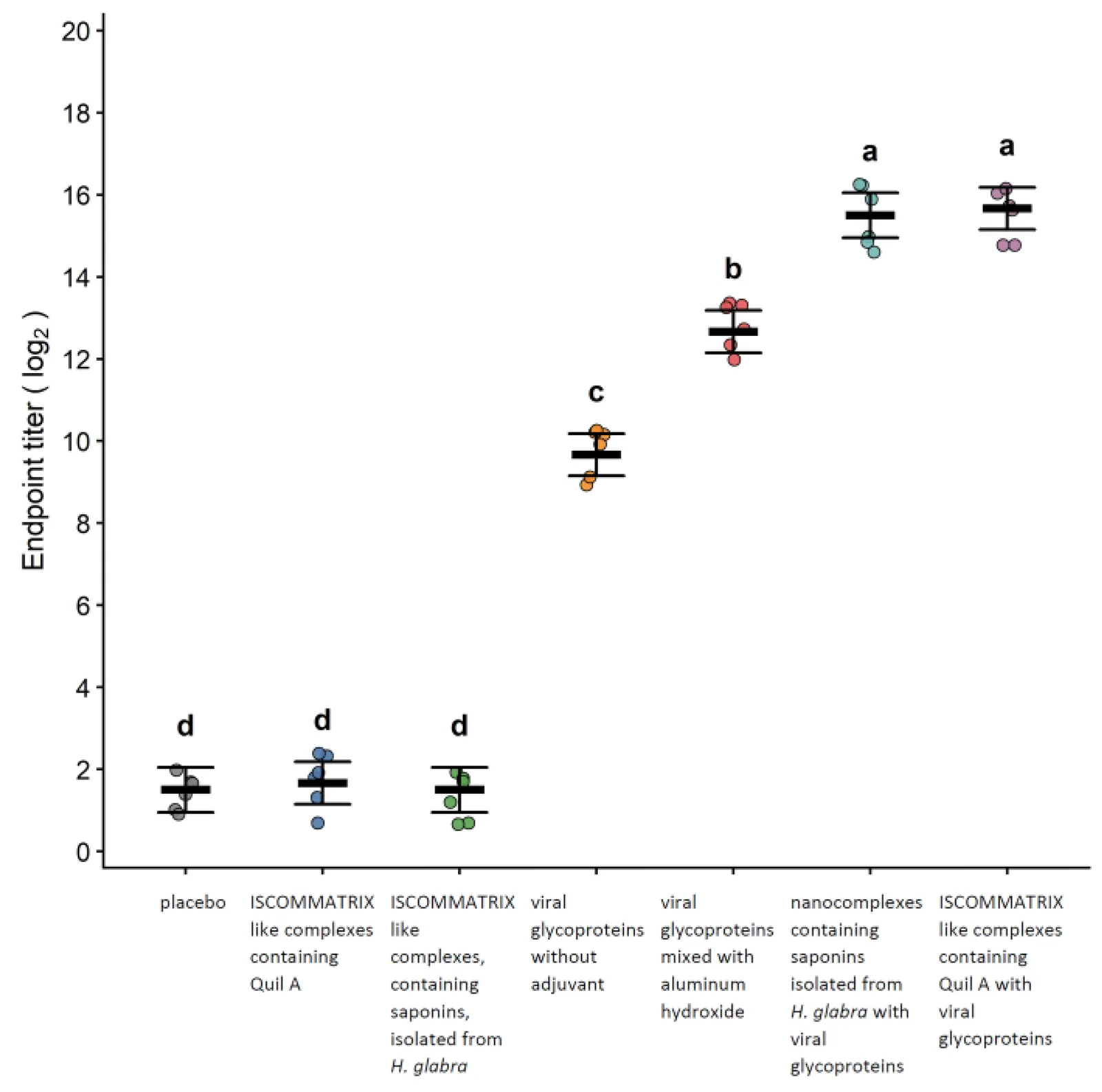
Induction of virus neutralization immune response.

The data presented are the average results from six separate mice. Different lowercase letters indicate statistically significant differences among groups according to one-way ANOVA followed by Tukey’s multiple comparison test (*p* < 0.05). Groups sharing at least one letter are not significantly different.

The robust neutralizing activity observed in animals immunized with the nanocomplex formulations, together with the high antigen-specific antibody titers, indicates that these formulations effectively promote the generation of functional antibody responses. This enhanced neutralizing capacity may be associated with improved antigen presentation and the particulate nature of the nanocomplexes, which facilitate antigen uptake and immune activation.

Overall, these findings demonstrate that the incorporation of influenza virus glycoproteins with *H. glabra*-based supramolecular nanocomplexes enhances the induction of functional virus-neutralizing antibodies. Although protective efficacy was not evaluated in a viral challenge model, the observed neutralizing antibody responses support the potential of these nanocomplexes as promising adjuvant systems for influenza vaccine development.

### 2.4. Induction of Cellular Immune Responses

The capacity of nanocomplexes with *H. glabra* saponins to stimulate cellular immunity was evaluated in a murine model. Three weeks after a single subcutaneous immunization, the production of Th1-associated cytokines (IFN-γ, IL-2) and Th2-associated cytokines (IL-4, IL-10) were quantified by ELISA.

As shown in Figure 5, nanocomplex-based formulations induced significantly higher cytokine levels compared with micellar antigen formulations adjuvanted with aluminum hydroxide.

**Figure 5:**
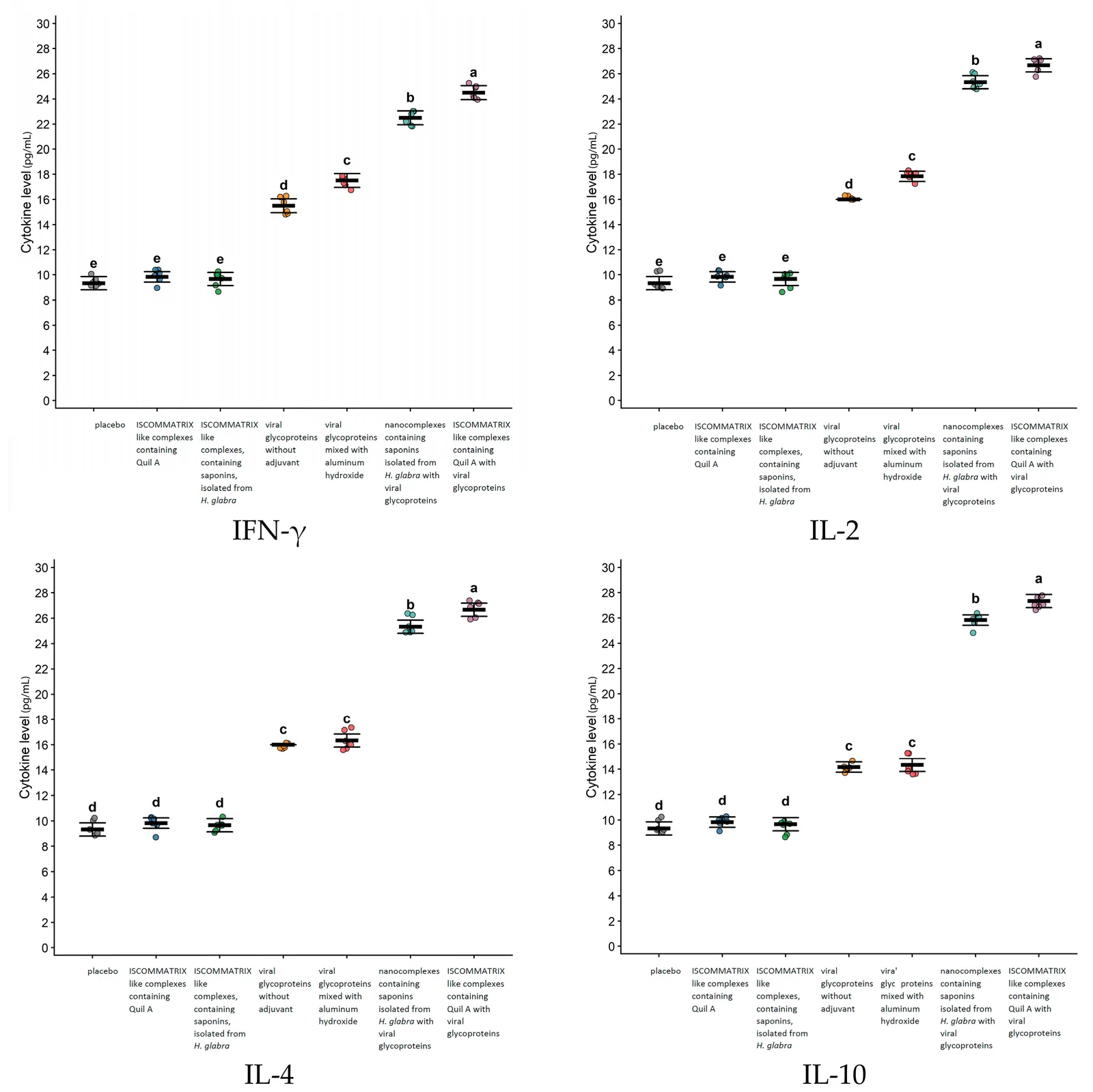
Induction of cellular immune responses by nanocomplexes of *H. glabra* saponins with purified influenza virus glycoproteins (HA/NA).

The data presented are the average results from six separate mice. Different lowercase letters indicate statistically significant differences among groups according to one-way ANOVA followed by Tukey’s multiple comparison test (*p* < 0.05). Groups sharing at least one letter are not significantly different.

Enhanced cytokine production indicates a stronger activation of antigen-specific T cell responses. Importantly, the magnitude of both Th1 and Th2 cytokine responses with nanocomplexes containing *H.glabra* saponins was comparable to that observed with ISCOMMATRIX-like complexes, suggesting that *H. glabra* saponins retain the potent immunostimulatory properties characteristic of classical saponin adjuvants.

The simultaneous induction of IFN-γ and IL-2, together with IL-4 and IL-10, indicates a balanced Th1/Th2 immune profile. Such a response is considered advantageous for vaccine efficacy, as Th1 responses support cellular immunity and viral clearance, while Th2 responses promote antibody production and humoral immunity.

Overall, these results indicate that *H. glabra* saponin-based nanocomplexes effectively stimulate both arms of the adaptive immune system, eliciting cellular responses comparable to those of previously described ISCOMMATRIX-like nanosystems, while *H. glabra* saponin-containing nanocomplexes potentially provide an improved safety profile.

## 3. Discussion

The growing interest in nanoparticle-based adjuvants reflects a broader transition in vaccinology toward platforms that integrate efficient antigen delivery with active immune modulation [23,24,25,26]. In the present study, *H. glabra*-derived saponins spontaneously self-assembled with phospholipids and cholesterol into stable nanocomplexes that exhibited favorable physicochemical characteristics, low in vitro cytotoxicity, and enhanced antigen-specific, virus-neutralizing antibody responses. These findings support the potential of plant-derived saponin nanocomplexes as promising platforms for vaccine adjuvant development.

The enhanced immunogenicity observed in this study is likely attributable to the combined effects of nanoparticle-mediated antigen delivery and the intrinsic immunostimulatory activity of saponins. Nanoparticle size, surface charge, and morphology are known to influence antigen uptake by antigen-presenting cells, lymphatic trafficking, and intracellular antigen processing [23,24,25,26], whereas saponins interact with membrane cholesterol and activate innate immune pathways that promote adaptive immune responses [8,9,10,23]. These complementary mechanisms are likely to act synergistically; however, the molecular basis of immune activation by *H. glabra*-derived saponins remains to be elucidated.

The enhanced antibody responses observed for the *H. glabra* nanocomplexes are consistent with previous studies demonstrating the potent adjuvant activity of triterpenoid saponins from *Quillaja saponaria* and other medicinal plants. Although direct comparisons between studies are limited by differences in antigen composition, formulation, immunization protocols, and experimental models, the magnitude of the humoral immune responses observed in the present study support the potential of *H. glabra* as an alternative botanical source of immunostimulatory saponins for nanoparticle vaccine formulations.

Comparison with the established ISCOMMATRIX-like platform provides an important benchmark for interpreting the present findings. Under the experimental conditions, the *H. glabra*-derived nanocomplexes exhibited physicochemical characteristics and immunological activity comparable to those of ISCOMMATRIX-like complexes. Nevertheless, these observations should not be interpreted as demonstrating equivalence between the two systems. ISCOMMATRIX-like formulations have undergone extensive optimization with respect to composition, stability, manufacturing consistency, and safety, whereas the nanocomplexes described here represent an early-stage proof-of-concept formulation requiring further optimization and validation.

An important consideration is that the investigated nanocomplexes were prepared from a naturally occurring mixture of *H. glabra* saponins rather than purified individual compounds. Although this structural diversity may contribute to the overall immunological properties of the formulation, it also limits identification of the individual saponins responsible for the observed biological effects. Further chromatographic isolation, structural characterization, and biological evaluation of individual saponins will, therefore, be necessary to establish structure–activity relationships, improve formulation standardization, and identify the principal immunologically active constituents.

Several limitations should be considered when interpreting the present findings. The study primarily evaluated humoral immune responses, whereas cellular immunity, cytokine production, and the molecular mechanisms of immune activation were not comprehensively investigated. In addition, antigen release kinetics and protective efficacy in a viral challenge model were not assessed. Although the nanocomplexes exhibited favorable in vitro cytotoxicity and were well tolerated during immunization, comprehensive preclinical safety evaluation, including dose-escalation studies, local reactogenicity, systemic toxicity, and histopathological assessment, remain necessary before clinical translation. Further optimization of formulation stability, scalability, manufacturing reproducibility, and long-term storage characteristics will also be essential for future development.

Overall, the present findings demonstrate that *H. glabra*-derived saponins can self-assemble into bioactive nanocomplexes capable of enhancing antigen-specific and virus-neutralizing antibody responses while maintaining favorable physicochemical characteristics and low in vitro cytotoxicity. Collectively, these results identify *H. glabra* as a promising botanical source of saponins for the development of next-generation nanoparticle vaccine adjuvants, and provide a foundation for further mechanistic and preclinical investigations.

## 4. Materials and Methods

### 4.1. Reagents

All chemicals and reagents were of analytical grade and used as received without further purification. Quil A adjuvant (Vac-quil-5) was obtained from InvivoGen (San Diego, CA, USA), and aluminum hydroxide gel (Alhydrogel^®^, 2%) was purchased from Croda International (Snaith, UK). Egg yolk-derived phosphatidylcholine, cholesterol (≥98% purity), and *N*-decanoyl-*N*-methylglucamine (MEGA-10) were purchased from Sigma-Aldrich (St. Louis, MO, USA).

Horseradish peroxidase (HRP)-conjugated secondary antibodies specific for mouse IgG, IgM, IgA, IgG1, IgG2a, and IgG2b, together with ELISA kits for IL-2, IL-4, IL-10, and IFN-γ, were obtained from Thermo Fisher Scientific (Invitrogen™, Waltham, MA, USA).

### 4.2. Viruses and Antigens

Influenza A virus strains A/Almaty/8/98 (H3N2) and the oseltamivir-resistant pandemic strain A/Vladivostok/2/09 (H1N1) were obtained from the Research and Production Center for Microbiology and Virology (Almaty, Kazakhstan).

Virus propagation, purification, concentration, and isolation of viral glycoproteins were performed as previously described [27,28,29]. The purified viral glycoproteins were subsequently used as antigens in all immunological experiments.

The herb of *H. glabra* was purchased from HORST, Barnaul, Russia. A voucher specimen (No. Hg-C19-1926) was deposited for the study of the biological properties of its saponins.

Saponins from *H. glabra* were isolated and purified according to a previously reported procedure with minor modifications [30,31]. Briefly, dried aerial plant material was extracted with aqueous ethanol, after which the solvent was removed under reduced pressure to obtain a crude extract. The saponin-rich fraction was subsequently purified by preparative high-performance liquid chromatography (prep-HPLC) using a Shimadzu preparative chromatography system (Shimadzu Corporation, Kyoto, Japan). The purified fraction was collected, concentrated under reduced pressure, and dried to yield purified *H. glabra* saponins, which were subsequently used for the preparation of nanoparticulate formulations. The chromatographic profiles of the purified *H. glabra* saponins and the proposed structures of the identified saponins are presented in the Supplementary Materials (Figure S1, Table S1). Chemically, these compounds are classified as members of the GOTCAB group (**G**lucuronide **O**leanane-type **T**riterpenoid **C**arboxylic **A**cid 3,28-**B**idesmosides).

### 4.3. Preparation of ISCOM-like Nanoparticles

ISCOMMATRIX-like nanoparticles were prepared by a detergent dialysis method as previously described [15,16,17,18] with minor modifications. Briefly, egg yolk phosphatidylcholine (1 mg), cholesterol (1 mg), purified *H. glabra* saponins or Quil A (1 mg) were mixed in phosphate-buffered saline (PBS, pH 7.4) containing 4% (*w*/*v*) *N*-decanoyl-*N*-methylglucamine (MEGA-10) to a final volume of 1 mL. The suspension was gently stirred until complete solubilization and was subsequently sonicated for 15 s to obtain a homogeneous mixture.

The detergent was removed by dialysis against 1 L of PBS (pH 7.4) at 4 °C for 12 h using dialysis tubing with a molecular weight cut-off (MWCO) of 3.5–14 kDa. The dialysis buffer was replaced twice during the procedure to ensure efficient detergent removal and promote spontaneous self-assembly of ISCOMMATRIX-like nanoparticles. The resulting nanoparticle suspension was extruded through 0.22-μm polycarbonate membranes (Whatman, Maidstone, UK) to obtain a uniform particle size distribution.

All nanoparticle formulations were prepared independently in three separate batches under identical experimental conditions.

### 4.4. Characterization of Nanoparticles

The hydrodynamic diameter, polydispersity index (PDI), and zeta potential of the nanoparticles were measured by dynamic light scattering using a Zetasizer Nano ZS (Malvern Panalytical, Malvern, UK) [32]. Measurements were performed in triplicate for each independently prepared batch, and the results are expressed as mean ± standard deviation (SD).

Nanoparticle morphology was examined by transmission electron microscopy (TEM) using a JEOL JEM-1400 transmission electron microscope (JEOL Ltd., Tokyo, Japan) operated at an accelerating voltage of 80 kV, following negative staining [33].

Viral glycoproteins were added to the nanocomplexes, followed by gentle mixing for 5 min to allow antigen association. The encapsulation efficiency (EE) of influenza hemagglutinin was determined by separating free and nanoparticle-associated antigen via high-speed centrifugation. The concentration of unbound antigen in the supernatant was quantified using a bicinchoninic acid (BCA) protein assay. Encapsulation efficiency was calculated using the following equationEE (%) = [(Total antigen − Free antigen)/Total antigen] × 100 [34,35].

Only nanoparticle batches exhibiting less than 10% variation in particle size and zeta potential were considered acceptable and were used for subsequent in vitro and in vivo experiments.

### 4.5. Cytotoxicity Assay

Cytotoxicity was evaluated using Caco-2, HT-29, and MDCK cell lines. Cells were seeded into 96-well plates and allowed to adhere overnight under standard culture conditions. Cell viability following exposure to serial dilutions of the formulations for 24–72 h was determined using the MTT assay as previously described [36,37]. Briefly, after incubation with the test formulations, MTT solution (0.5 mg/mL) was added to each well, and the resulting formazan crystals were dissolved in dimethyl sulfoxide (DMSO). Absorbance was measured at 570 nm using a microplate reader. Cell viability was expressed as a percentage of the untreated control, and the half-maximal cytotoxic concentration (CC_50_) was calculated by nonlinear regression analysis.

### 4.6. Immunization Protocol

Male BALB/c mice (4–5 weeks old; *n* = 6 per group) were obtained from a certified breeding facility and maintained under specific pathogen-free conditions (22 ± 2 °C, 50–60% relative humidity, 12 h light/dark cycle) with ad libitum access to food and water. Animals were acclimatized for at least 7 days before the study. All procedures were approved by the institutional animal ethics committee and performed in accordance with relevant national and international guidelines [38,39,40].

Mice were randomly assigned to experimental groups using a computer-generated randomization schedule. Investigators responsible for immunological analyses and statistical evaluation were blinded to group allocation until completion of the study.

Animals were assigned to seven experimental groups receiving (i) placebo (PBS); (ii) Quil A saponin alone; (iii) purified *H. glabra* saponins alone; (iv) influenza viral glycoproteins alone; (v) influenza viral glycoproteins adsorbed to aluminum hydroxide; (vi) influenza viral glycoproteins formulated with *H. glabra* saponin nanocomplexes; (vii) ISCOMMATRIX-like complexes with influenza viral glycoproteins and Quil A. Immunizations were administered subcutaneously in a total volume of 200 μL. Each vaccine dose contained 10 μg of influenza viral glycoproteins, while ISCOMMATRIX-like formulations contained 2 μg of Quil A or *H. glabra* saponins per mouse. Animals were immunized according to a prime–boost regimen, with a booster dose administered 21 days after the primary immunization (Figure 6).

Blood samples were collected three weeks after booster immunization by retro-orbital venous plexus puncture under light isoflurane anesthesia. Serum was separated by centrifugation and stored at −20 °C until immunological analyses.

Animals were monitored daily throughout the study for general health and signs of adverse reactions. Pre-specified exclusion criteria included unrelated illness, technical failure during sample collection, or insufficient sample volume. Humane endpoints were defined as >20% body weight loss, markedly reduced mobility, or signs of severe distress.

### 4.7. Enzyme-Linked Immunosorbent Assay (ELISA)

Serum samples were collected three weeks after the primary immunization and three weeks after the booster immunization. Antigen-specific antibody responses were determined by indirect ELISA as previously described [41,42].

Briefly, 96-well microplates were coated overnight at 4 °C with purified influenza viral glycoproteins, blocked with blocking buffer, and incubated with serial two-fold dilutions of serum samples. After washing, horseradish peroxidase (HRP)-conjugated secondary antibodies specific for mouse IgG, IgM, IgA, IgG1, IgG2a, or IgG2b were added. The reaction was developed using o-phenylenediamine dihydrochloride (OPD) substrate and stopped with 2 M sulfuric acid. Absorbance was measured at 490 nm using a Tecan Infinite^®^ 200 PRO microplate reader (Tecan Group Ltd., Männedorf, Switzerland). All samples were analyzed in duplicate.

Antibody titers were defined as the highest serum dilution producing an optical density greater than twofold that of the negative control.

Serum concentrations of IL-2, IL-4, IL-10, and IFN-γ were quantified using commercial sandwich ELISA kits (Thermo Fisher Scientific, Waltham, MA, USA) according to the manufacturer’s instructions. Cytokine measurements were performed in duplicate.

### 4.8. Microneutralization Assay

Virus-neutralizing antibody titers were determined using MDCK cells as previously described [43]. Heat-inactivated serum samples were serially two-fold diluted and incubated with 100 TCID_50_ of influenza virus for 1 h at 37 °C. The virus–serum mixtures were then added to confluent MDCK cell monolayers cultured in 96-well plates and incubated at 37 °C in a humidified atmosphere containing 5% CO_2_ for 48–72 h.

Cytopathic effects (CPE) were evaluated by light microscopy. Neutralizing antibody titers were expressed as the reciprocal of the highest serum dilution that completely inhibited virus-induced cytopathic effects in at least 50% of replicate wells.

### 4.9. Statistical Analysis

Data are presented as the mean ± standard deviation (SD). Statistical analyses were performed using GraphPad Prism version 10.2.3 (GraphPad Software, San Diego, CA, USA).

Data normality was assessed using the Shapiro–Wilk test. Comparisons between two groups were performed using an unpaired two-tailed Student’s *t*-test, whereas comparisons among multiple groups were analysed using one-way analysis of variance (ANOVA) followed by Tukey’s multiple-comparison test. A value of *p* < 0.05 was considered statistically significant [44].

No data were excluded unless predefined technical exclusion criteria were met. Individual animals were considered the experimental units for all statistical analyses.

## Figures and Tables

**Figure 1:**
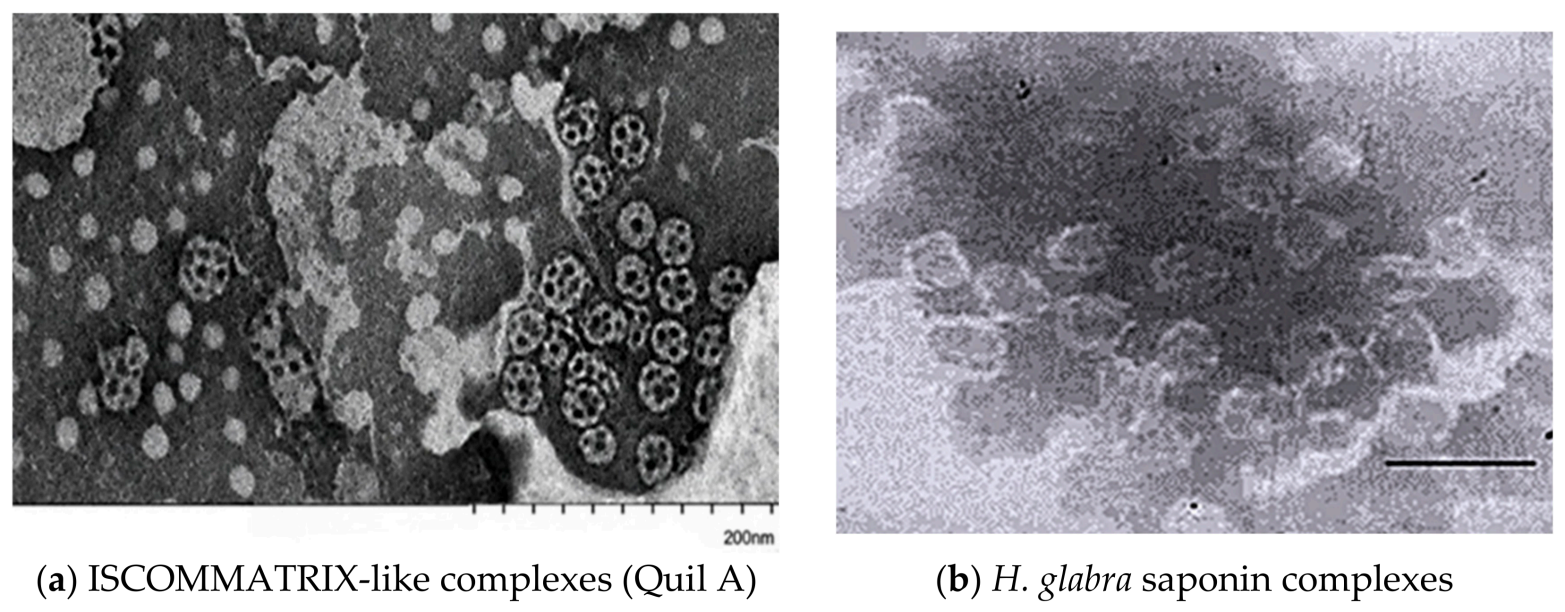
Supramolecular saponin-based nanocomplexes visualized by transmission electron microscopy using negative staining at 100,000× magnification.

**Figure 2:**
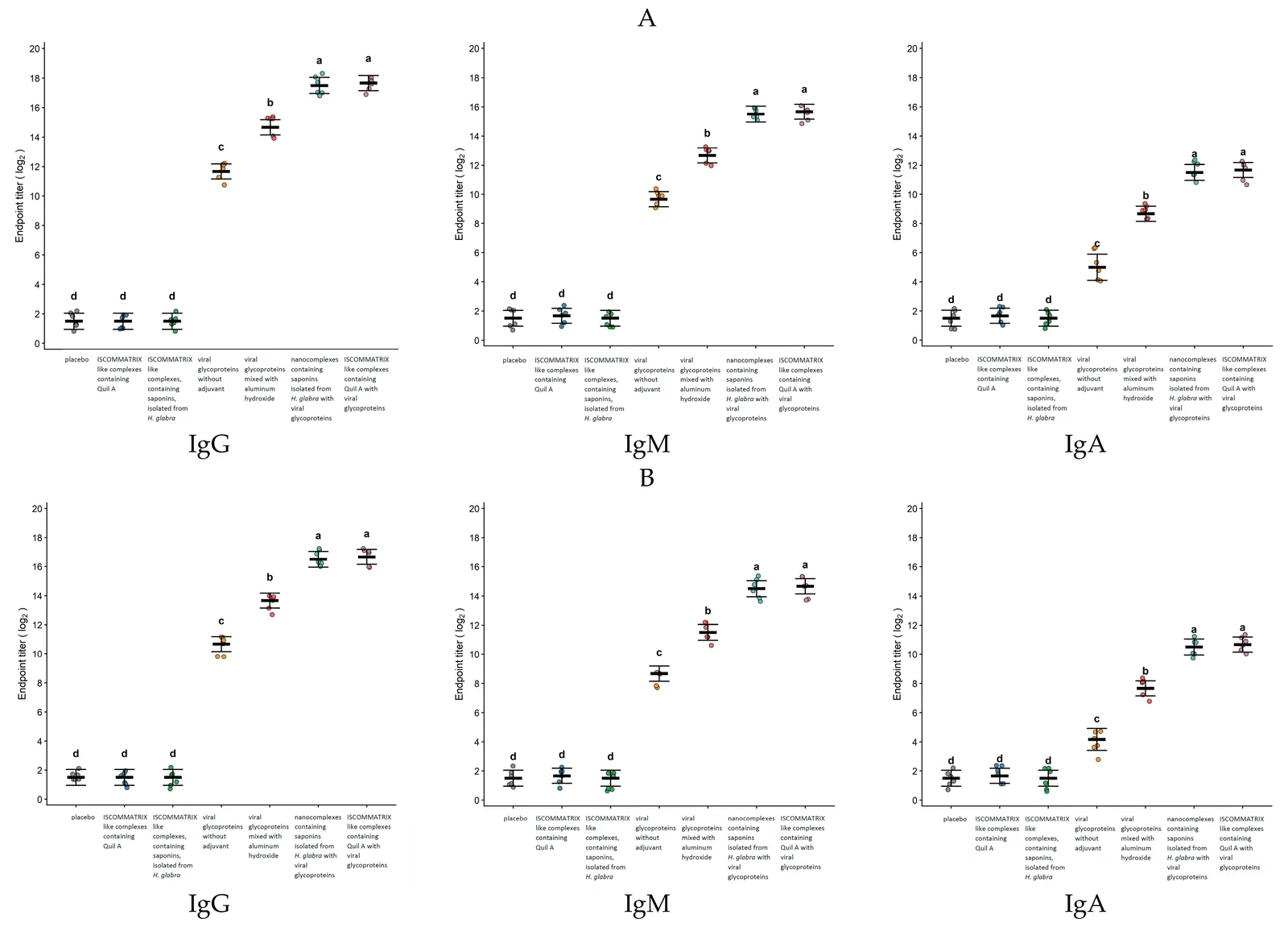
Immunostimulatory activity of nanocapsulated adjuvant containing saponins isolated from *the H. glabra* plant on the model of influenza viral glycoproteins (HA/NA). (**A**)—human influenza virus, strain A/Almaty/8/98 (H3N2); (**B**)—Tamiflu-resistant pandemic variant influenza virus A/Vladivostok/2/09 (H1N1).

**Figure 6:**
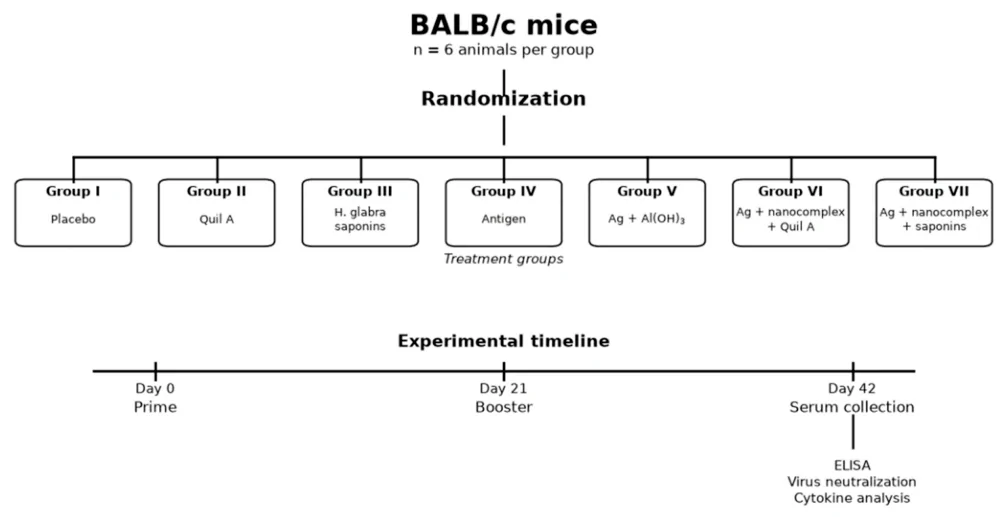
Immunization scheme.

**Table 1:** Physicochemical characteristics of supramolecular formulations.

Formulation	Size (nm)	PDI	Zeta Potential (mV)	Encapsulation Efficiency (%)
ISCOMMATRIX (Quil A)	75 ± 9.5	0.21 ± 0.08	−13.4 ± 5.6	92.3 ± 3.5
*H. glabra* saponin complexes	120.2 ± 11.1	0.35 ± 0.05	−32.6 ± 5.2	90.2 ± 4.1

Data are presented as mean ± SD from three independent preparations.

**Table 2:** Cytotoxicity of tested saponins in vitro.

Saponin	CC_50_ (µg/ml) MDCK *	CC_50_ (µg/ml) Caco2 *	CC_50_ (µg/ml) HT26 *
Quil A	28.13 ± 8.3	15.3 ± 2.8	18.25 ± 2.9
ISCOMMATRIX with Quil A	92.1 ± 6.8	105.2 ± 8.45	153.24 ± 15.3
Saponins from Herniaria glabra	45.3 ± 4.7	33.41 ± 5.31	36.4 ± 5.1
Complexes with saponins from Herniaria glabra	102.7 ± 7.2	154.2 ± 9.1	203.4 ± 15.24

* Mean values and SDs from triplicate assays.

## Data Availability

All the data generated or analyzed during this study has been included in the published article.

## Supplementary Materials

The following supporting information can be downloaded at https://www.mdpi.com/article/10.3390/molecules31193523/s1: Figure S1: Analytical HPLC chromatogram of the saponin-rich fraction obtained from *H. glabra (3% of extract)*. The chromatographic profile shows the major components of the fraction used for preparation of the nanocomplexes; Table S1: Structure *Herniaria saponins*.
