# Supplementary material for: Evaluation of the Adjuvant Activity of Herniaria glabra Saponin-Based Nanoadjuvants
Source: Molecules. 2026 Oct 2;31(19):3523. doi: 10.3390/molecules31193523 (PMC13666411; doi:10.3390/molecules31193523)
Supplement: Supplementary file 1 [file molecules-31-03523-s001.zip › molecules-4553172-supplementary.pdf]

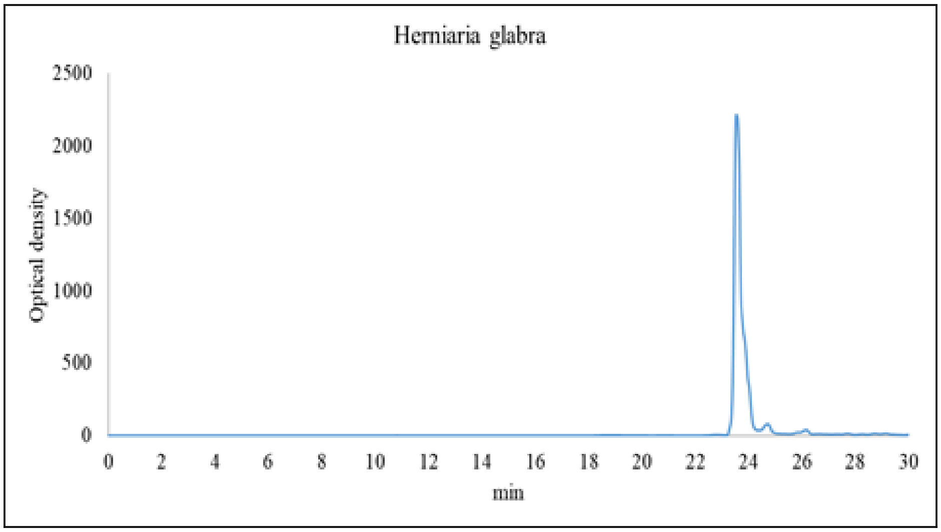

Figure S1. Analytical HPLC chromatogram of the saponin-rich fraction obtained from *H. glabra (3% of extract)*. The chromatographic profile shows the major components of the fraction used for preparation of the nanocomplexes.

Table S1. Structure Herniaria saponins

Saponin	Structure	Describtion
Herniaria Saponin I	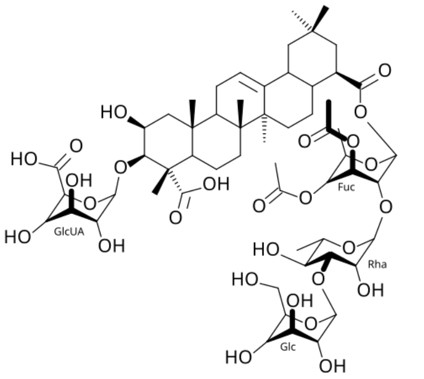	Saponin glycoside in *Herniaria glabra*. created with bkchem+inkscape Source of information: Anwer Basha, Viqar U Ahmad: *Spectroscopic Data of Saponins.* CRC Press, 2000. ISBN: 0849394910
Herniaria saponin H	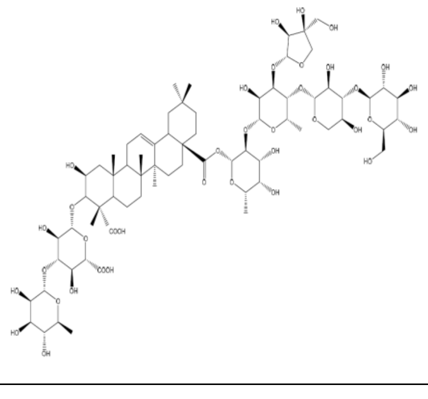	Peeters L, Foubert K, Breynaert A, Schreurs G, Verhulst A, Pieters L, Hermans N. Effects of medicagenic acid metabolites, originating from biotransformation of an Herniaria hirsuta extract, on calcium oxalate crystallization in vitro. J Ethnopharmacol. 2022 Mar 1;285:114860. doi: 10.1016/j.jep.2021.114860
